# Pulmonary toxicity of well-dispersed cerium oxide nanoparticles following intratracheal instillation and inhalation

**DOI:** 10.1007/s11051-015-3249-1

**Published:** 2015-11-13

**Authors:** Yasuo Morimoto, Hiroto Izumi, Yukiko Yoshiura, Taisuke Tomonaga, Takako Oyabu, Toshihiko Myojo, Kazuaki Kawai, Kazuhiro Yatera, Manabu Shimada, Masaru Kubo, Kazuhiro Yamamoto, Shinichi Kitajima, Etsushi Kuroda, Kenji Kawaguchi, Takeshi Sasaki

**Affiliations:** University of Occupational and Environmental Health, 1-1 Iseigaoka, Yahata-nishi-ku, Kitakyushu, Fukuoka 807-8555 Japan; Hiroshima University, Higashi, Hiroshima Japan; National Institute of Advanced Industrial Science and Technology (AIST), 1-1-1 Higashi, Tsukuba, Ibaraki 305-8565 Japan; National Sanatorium Hoshizuka Keiaien, 4204 Hoshizuka-cho, Kanoya, Kagoshima 893-8502 Japan; Laboratory of Vaccine Science, WPI Immunology Frontier Research Center, Osaka University, 6F IFReC Research Building, 3-1 Yamada-oka, Suita, Osaka 565-0871 Japan

**Keywords:** Cerium dioxide, Nanoparticle, Intratracheal instillation, Pulmonary inflammation, Chemokine, Environmental and health effects

## Abstract

We performed inhalation and intratracheal instillation studies of cerium dioxide (CeO_2_) nanoparticles in order to investigate their pulmonary toxicity, and observed pulmonary inflammation not only in the acute and but also in the chronic phases. In the intratracheal instillation study, F344 rats were exposed to 0.2 mg or 1 mg of CeO_2_ nanoparticles. Cell analysis and chemokines in bronchoalveolar lavage fluid (BALF) were analyzed from 3 days to 6 months following the instillation. In the inhalation study, rats were exposed to the maximum concentration of inhaled CeO_2_ nanoparticles (2, 10 mg/m^3^, respectively) for 4 weeks (6 h/day, 5 days/week). The same endpoints as in the intratracheal instillation study were examined from 3 days to 3 months after the end of the exposure. The intratracheal instillation of CeO_2_ nanoparticles caused a persistent increase in the total and neutrophil number in BALF and in the concentration of cytokine-induced neutrophil chemoattractant (CINC)-1, CINC-2, chemokine for neutrophil, and heme oxygenase-1 (HO-1), an oxidative stress marker, in BALF during the observation time. The inhalation of CeO_2_ nanoparticles also induced a persistent influx of neutrophils and expression of CINC-1, CINC-2, and HO-1 in BALF. Pathological features revealed that inflammatory cells, including macrophages and neutrophils, invaded the alveolar space in both studies. Taken together, the CeO_2_ nanoparticles induced not only acute but also chronic inflammation in the lung, suggesting that CeO_2_ nanoparticles have a pulmonary toxicity that can lead to irreversible lesions.

## Introduction

Although cerium (Ce) is a rare earth lanthanide metal, there are large amounts of it in the earth’s crust. CeO_2_ is widely used in abrasives for optical glass and glass substrates for liquid crystal displays and hard disks. It has also been used more recently as a fuel additive to improve combustion processes, and it works as a catalyst to increase fuel efficiency and reduce particle emissions in exhaust gas. The Organization for Economic Co-operation and Development (OECD) has launched a sponsorship program for the testing of manufactured nanomaterials, and 14 different nanomaterials, including CeO_2_, have a high-priority for evaluation. CeO_2_ is expected to be a nanomaterial in high demand, but the effects of CeO_2_ on the lung are not fully known.

In lung disorders caused by dust, phagocytosis of dust induces infiltration of neutrophils and alveolar macrophages, and persistent or progressed inflammation is likely to cause lung injury and lead to irreversible changes, such as fibrosis and tumors (Nishi et al. 2009; Borm and Driscoll 1996; Kim et al. 2007). Persistent inflammation has been reported in an animal exposure model using asbestos and silica as materials known to have high toxicity (Fubini and Hubbard 2003; Schins 2002). As for CeO_2_ nanoparticles, some intratracheal instillation and inhalation studies have shown pulmonary inflammation, suggesting that CeO_2_ nanoparticles may have harmful effects on humans. Most of those reports show acute pulmonary inflammation, and there were no reports evaluating chronic responses such as persistent inflammation. Studies of crystalline silica, a material with high toxicity, have revealed an exacerbation of inflammation in the lung 1 or 2 months postexposure, and inhalation of crystalline silica induced pulmonary damage in rats at 10 weeks and 16 weeks after exposure (Sellamuthu et al. 2011; Langley et al. 2004). Considering that the pulmonary toxicity of crystalline silica was induced in the chronic phase, it is important to evaluate its harmful effects after sufficient recovery time. Therefore, in order to explore the pulmonary toxicity of CeO_2_ nanoparticles we performed intratracheal instillation and inhalation studies with more than 3 months of observation time and examined the pulmonary inflammation and fibrosis as the endpoints of toxicity.

## Materials and methods

### Sample preparation of CeO_2_ nanoparticle suspensions

Commercial cerium dioxide (CeO_2_) nanoparticles (Wako Chemical, Ltd.) were dispersed in deionized endotoxin-free water. The purity of the CeO_2_ nanoparticles as measured by the supplier was 99.9 wt%. Supernatants obtained by a combination of 2 h ultrasonic homogenizing (Branson 5510J-MT, 42 kHz 180 W) and 20,000×*g*—30 min centrifugation (Hitachi Koki Co., Ltd., CF16RX2) were in stable suspension without agglomerated coarse particles larger than 1 μm. The detailed procedures are explained in our previous report (Kubo et al. 2014). The physicochemical properties of CeO_2_ nanoparticles are shown in Table 1.

**Table 1:** Physicochemical properties of CeO_2_ used in the experiment

Physicochemical properties	CeO_2_ nanoparticle
Chemical formula	CeO_2_
Product name and manufacturer	Cerium oxide (IV) Nanoparticle Wako Chemical, Ltd.
Primary diameter	7.8 nm
Specific surface area (BET)	101 m^2^/g
Shape	Irregular shape
Secondary particle diameter (DLS; number based)	10.0 nm
Crystal structure	Fluorite structure
Purity	99.9 %
Bulk density	7.216 g/cm^3^
Solubility	Insoluble in water

**Fig. 1:**
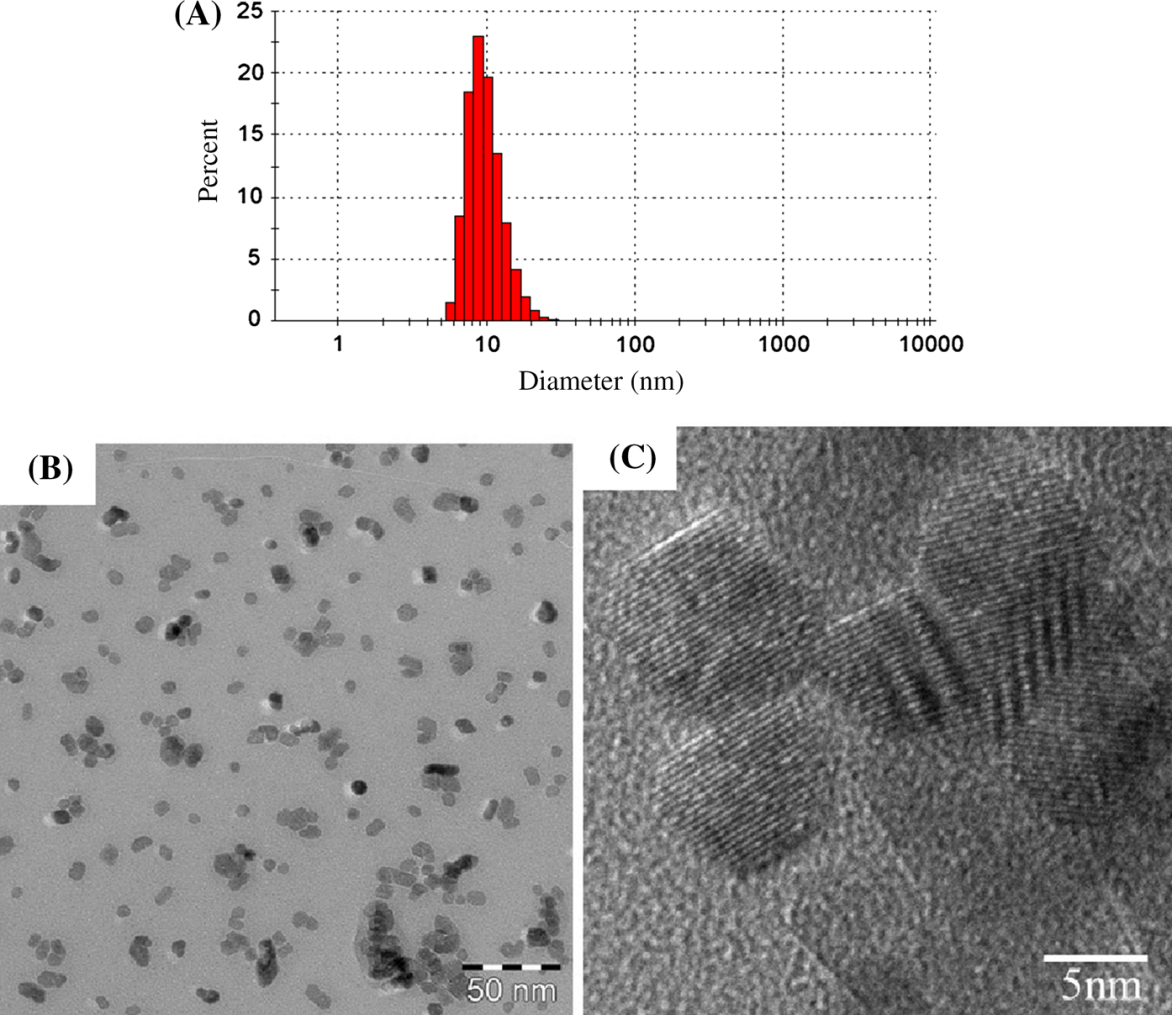
CeO_2_ nanoparticle agglomerates suspended in distilled water. **a** The number-based average particle size measured by dynamic light scattering is 10.0 nm. CeO_2_ nanoparticles were well-dispersed in suspensions. **b** CeO_2_ nanoparticles by transmission electron microscopy. **c** Magnified images of CeO_2_ nanoparticles

The number-based average particle size measured by dynamic light scattering (DLS, Zeta sizer nano-ZS, Malvern, England) was 10.0 nm, as shown in Fig. 1a. The CeO_2_ nanoparticle suspensions were observed by transmission electron microscope (TEM, EM922, Carl Zeiss, Germany) at an accelerating voltage of 200 kV. TEM specimens were prepared by dropping suspensions on TEM grids with carbon support films and being dried. TEM images of the CeO_2_ nanoparticle suspensions are shown in Fig. 1b, c. Most of the CeO_2_ particles were mono-dispersed, and some made up aggregates of between 20 and 30 nm in size. The primary CeO_2_ particle size was approximately 8 nm, which is almost the same as the value measured by DLS. As can be seen in the high-resolution image, the CeO_2_ particles had a clear crystalline form, and there was no damage caused by the preparation processes, meaning that the CeO_2_ primary particles were dispersed well and stably in the water without significant agglomeration.

### Animals

Male Fischer 344 rats (9–11 weeks old) were purchased from Charles River Laboratories International, Inc. (Japan). The animals were kept in the Laboratory Animal Research Center of the University of Occupational and Environmental Health for 2 weeks with access to free-feeding of commercial diet and water. All procedures and animal handling were done according to the guidelines described in the Japanese Guide for the Care and Use of Laboratory Animals as approved by the Animal Care and Use Committee, University of Occupational and Environmental Health, Japan.

### Intratracheal instillation of nanomaterial

The CeO_2_ nanoparticles were suspended with 0.4 ml distilled water. 0.2 mg (0.8 mg/kg) or 1 mg (4 mg/kg) of CeO_2_ nanoparticles was intratracheally instilled once to rats (12 weeks old). The negative control groups received distilled water. Animals were dissected at 3 days, 1 week, 1, 3, and 6 months after the instillation.

### Inhalation of nanomaterial

The inhalation test was conducted by supplying CeO_2_ aerosol particles at two concentrations. The particles were generated from the CeO_2_ nanoparticle suspension by the spray-drying method. The details of the experimental setup and conditions were similar to those in our previous study (Shimada et al. 2009; Kubo et al. 2014). In brief, an aerosol generation system consisting of a pressurized nebulizer and a drying section was connected to a whole body exposure chamber with rat cages. The CeO_2_ suspensions were prepared at concentrations of 3–5 mg/mL for a high-dose chamber and 0.6–0.8 mg/mL for a low-dose chamber, respectively. The CeO_2_ suspension was set in the nebulizer to be sprayed with compressed air at a flow rate of 40 L/min. The resulting droplets were successively passed through the drying section to remove the water from the droplets. The aerosol was diluted with clean air to ensure a necessary total airflow rate of 100 L/min, and fed through the exposure chamber for 6 h on each day of the inhalation test (a period of 4 weeks). The particle size distribution of the aerosols in the exposure chamber was measured twice per 0.5 h using a particle size spectrometer (model 1000XP WPS, MSP Corp., Shoreview, MN). The aerosol particles were also sampled outside of the chamber by an electrostatic precipitator for off-line analysis using TEM. The mass concentration of the aerosol in the chamber was determined by the gravimetrical method, i.e., the aerosol was admitted through fibrous filters, and the collected particles were weighed. The mass concentration was measured 3–5 times per day. After 4 weeks of exposure, the rats were dissected at 3 days, 1, and 3 months of recovery.

**Fig. 2:**
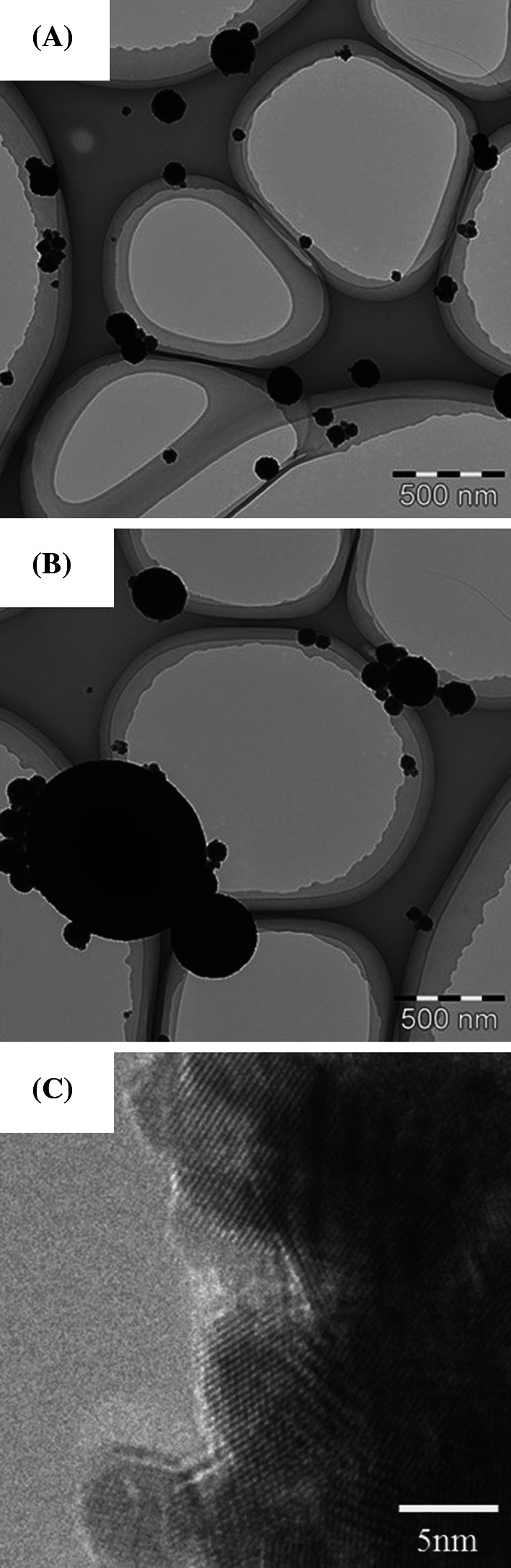
TEM images of inhaled CeO_2_ nanoparticles in exposure chambers. **a**, **b** CeO_2_ nanoparticles by transmission electron microscopy. **c** Magnified images of CeO_2_ nanoparticles. A clear crystal lattice image was observed. Inhaled CeO_2_ nanoparticles were well-dispersed in the exposure chamber

The CeO_2_ aerosols in both the low- and high-dose chambers had very stable particle size distribution for 6 h for each of the days of the entire period of the inhalation test. The average geometric mean diameters of the aerosol particles for the 4 weeks were 110 ± 12.5 nm (*N* = 480) for the high-dose chamber and 87.6 ± 7.9 nm (*N* = 480) for the low-dose chamber, respectively. The mass concentrations measured daily for the 4 weeks were 10.2 ± 1.38 mg/m^3^ (*N* = 82) for the high-dose chamber and 2.09 ± 0.29 mg/m^3^ (*N* = 80) for the low-dose chamber, respectively. TEM images of the CeO_2_ aerosols in the high-dose chambers are shown in Fig. 2a–c. The aerosols were aggregates, and their size was between 50 and 500 nm. The size of most of the aggregates was about 100 nm, which is in good agreement with the value measured by the particle size spectrometer. A high-resolution TEM image (Fig. 2c) shows a clear crystal lattice, and there was no degradation of the CeO_2_ particles in the aerosol generation process.

### Animals after the inhalation and intratracheal instillation studies

There were ten rats, divided into two subgroups of five animals each, in the control, low-dose and high-dose groups in each time course for lung tissue analysis. In the first subgroups, the lungs were inflated with physiological saline with 20 mL under a pressure of 20 cm water, and BALF was collected from whole lung divided two to three times. Between 15 and 18 mL of BALF was collected in collection tubes by free fall. In the second subgroups, the lungs were divided into right and left lungs. Analysis of cytokine was performed with the homogenized right lung, and histopathological evaluation was performed with the left lung inflated and fixed by 10 % formaldehyde.

### Analysis of inflammatory cells in BALF with cytospin

From 10 to 13 mL of BALF was centrifuged at 400 g at 4 °C for 15 min. The supernatant was transferred to a new tube and used for measuring the cytokines in the BALF. The pellets were washed by suspension with PMN Buffer (137.9 mM NaCl, 2.7 mM KCl, 8.2 mM Na_2_HPO_4_, 1.5 mM KH_2_PO_4_, 5.6 mM C_6_H_12_O_6_) and centrifuged at 400 g at 4 °C for 15 min. After the supernatant was removed, the pellets were resuspended with 1 mL of PMN Buffer. The cell number in the BALF was counted by Celltac (Nihon Kohden, Tokyo, Japan), and cells were splashed on a slide glass using cytospin. After the cells were fixed and stained with Diff-Quik (System Corporation, Hyogo, Japan), the number of neutrophils was counted by microscopic observation.

### Chemokines, lactate dehydrogenase (LDH), and heme oxygenase-1 (HO-1) measurement of BALF

The concentrations of Rat CINC-1 and Rat CINC-2α/β in the BALF and lung tissue were measured by ELISA kits, #RCN100, #RCN200, #RCN300 (R&D Systems, Minneapolis, MN), respectively. The concentrations of Rat HO-1 were measured by an ELISA kit, ADI-EKS-810A (Enzo Life Sciences, Farmingdale, NY), and the activity of released LDH was measured by a Cytotoxicity Detection Kit^PLUS^(LDH) (Roche Diagnostics GmbH, Mannheim, Germany). The LDH activity is determined in an enzymatic test (LDH-catalyzed conversion of lactate to pyruvate). All measurements were performed according to the manufacturer’s instructions.

### Histopathology

The lung tissue, which was inflated and fixed with 10 % formaldehyde under a pressure of 25 cm water, was embedded in paraffin, and 5 μm thick sections were cut from the lobe, then stained with hematoxylin and eosin.

### TEM experimental methods

Lung tissues were observed by TEM after the inhalation and intratracheal instillation studies. The method used for the TEM specimen preparation is described below. The lung tissues were fixed by a perfusion system using a 4 % paraformaldehyde solution, and were then post-fixed using a 1 % osmium tetroxide solution. They were subsequently dehydrated in ethanol, followed by embedding in epoxy resin. Ultrathin sections were cut with a diamond knife using microtomy. A part of the specimen was stained with a 2 % uranyl acetate solution and a mixed solution of 0.3 % lead nitrate and 0.3 % lead acetate, all at room temperature. Conventional TEM observation was performed with an H-7600 (Hitachi, Japan) at an accelerating voltage of 80 kV.

### Statistical analysis

Analysis of variance (ANOVA) and Dunnett’s test were applied where appropriate to determine individual differences using a computer statistical package (SPSS, SPSS Inc., Chicago, IL, U.S.A.).

## Results

### Intratracheal instillation study

#### Cell analysis in BALF

**Fig. 3:**
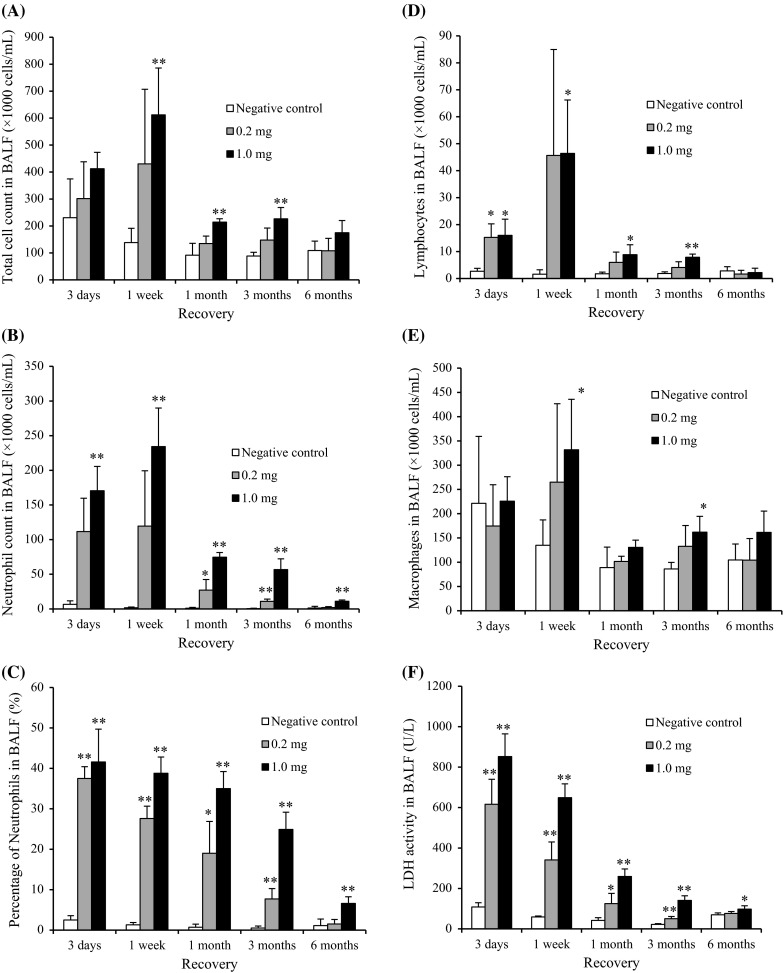

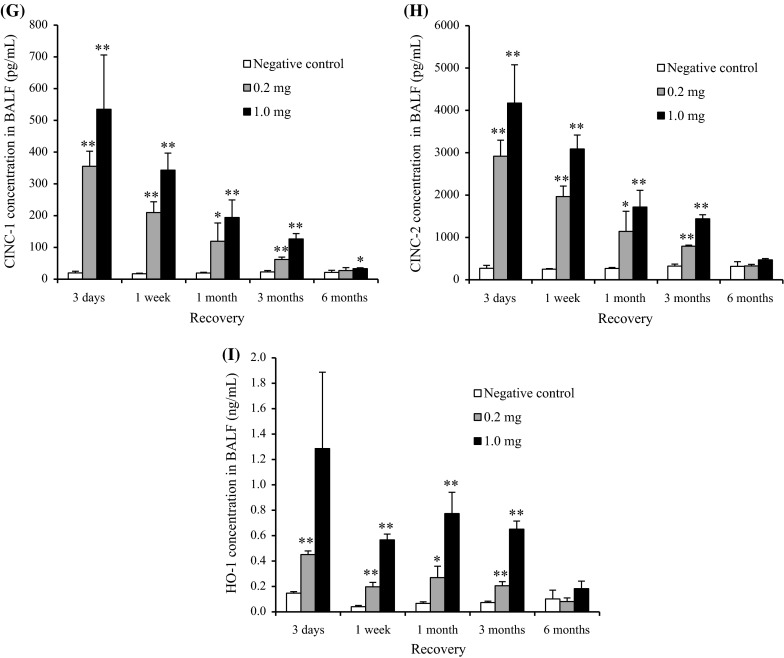
Cell analysis and cytokine concentration in BALF following intratracheal instillation of nanoparticles. **a** Total cell count in BALF. **b** Neutrophil count in BALF. **c** Percentage of neutrophils in BALF. **d** Lymphocyte count in BALF. **e** Alveolar macrophage count in BALF. **f** LDH activity in BALF. **g** Concentration of CINC-1 in BALF. **h** Concentration of CINC-2 in BALF. **i** Concentration of HO-1 in BALF. Intratracheal instillation of CeO_2_ nanoparticles induced persistent influx of inflammatory cells and expression of CINC-1, CINC-2, and HO-1 in BALF

Figure 3 shows the cellular analysis of the BALF following the intratracheal instillation of CeO_2_ nanoparticles. In comparison with the negative control, the total cell count in BALF was significantly and persistently higher in the 1 mg group from 1 week to 3 months postexposure. The neutrophil counts and percentage in the BALF were also persistently and dose-dependently high in the 0.2 mg and the 1 mg groups. The macrophage and lymphocyte counts in the BALF were high in the 1 mg group at 1 week and 3 months and from 3 days to 3 months postexposure, respectively. A persistent increase in the released LDH activity was observed in the 0.2 and 1 mg groups. The peak level of LDH was at 3 days, and it returned to nearly the level of the negative control according to a time course, although there was a significant difference of value between the negative and the 1 mg groups until 6 months postexposure.

#### CINC concentration in BALF

Figure 3g, h shows the concentrations of CINC-1 and CINC-2 in the BALF following the intratracheal instillation of CeO_2_ nanoparticles. The concentrations of CINC-1 and CINC-2 were persistently and dose-dependently high in both the 0.2 mg and the 1 mg groups. The maximum value of the CINC-1 and CINC-2 during the observation time was at 3 days, and it decreased gradually to nearly the negative control level at 6 months, as like the released LDH activity.

#### HO-1 concentration in BALF

The concentration of HO-1 in the 0.2 and 1 mg groups was significantly and persistently higher than that in the negative control group (Fig. 3i). As with the concentrations of CINC-1, CINC-2, and LDH, the peak value of HO-1 was at 3 days.

#### Histopathological changes in the lungs (Fig. 4; Table 2)

Particle-laden macrophages were distributed around the alveolar ducts and the surrounding alveolar spaces. There were many brown particles in the cytoplasm of the macrophages at 3 days after exposure, and the number of brown pigments decreased at 1 and 3 months after exposure. More macrophages were observed in the 1 mg instillation groups than in the 0.2 mg instillation groups. Moderate inflammatory changes were observed in the groups 3 days and 1 week after exposure, and the inflammation diminished 1 or 3 months after exposure. Cellular infiltrations and mild thickening of alveolar septa were observed at 3 days after exposure, and they decreased at 1 or 3 months after exposure.

**Fig. 4:**
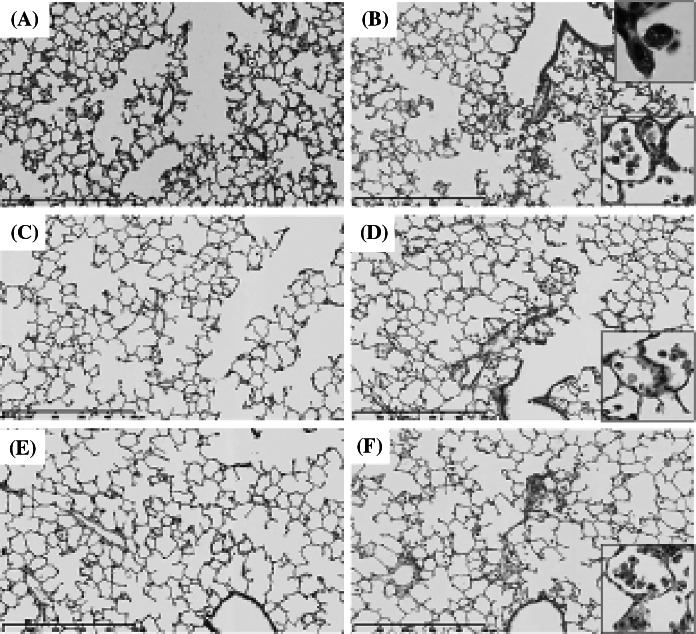
Hematoxylin and eosin staining of lung sections following intratracheal instillation of CeO_2_ nanoparticles. (×40, *inset* ×200). **a** Lung of negative control at 3 days postexposure, **b** 1 mg CeO_2_-exposed lung at 3 days postexposure. **c** Lung of negative control at 1 month postexposure, **d** 1 mg CeO_2_-exposed lung at 1 month postexposure. **e** Lung of negative control at 3 months postexposure, **f** 1 mg CeO_2_-exposed lung at 3 months postexposure. Persistent inflammation, mainly neutrophils and alveolar macrophages, was observed after intratracheal instillation of CeO_2_ nanoparticles

**Table 2:** Pathological features in the rat lung following intratracheal instillation of CeO_2_ nanoparticles

Pathological feature	3 days (*n* = 5)			1 week (*n* = 5)			1 month (*n* = 5)			3 months (*n* = 5)			6 months (*n* = 5)		
	Negative control	CeO_2_ 0.2 mg	CeO_2_ 1.0 mg	Negative control	CeO_2_ 0.2 mg	CeO_2_ 1.0 mg	Negative control	CeO_2_ 0.2 mg	CeO_2_ 1.0 mg	Negative control	CeO_2_ 0.2 mg	CeO_2_ 1.0 mg	Negative control	CeO_2_ 0.2 mg	CeO_2_ 1.0 mg
Macrophage infiltration in alveolar space	− to ±	±	+	− to ±	± to +	+	− to ±	± to +	+	− to ± to ±	+	+ to +	–	±	± to +
Neutrophil infiltration in alveolar space	−	−	− to ±	−	−	±	−	−	−	−	−	−	−	−	−
Infiltration in interstitial area	−	−	−	−	−	−	−	−	−	−	−	−	−	−	−
Fibrosis	−	−	−	−	−	−	−	−	−	−	−	−	−	−	−
Tumor	−	−	−	−	−	−	−	−	−	−	−	−	−	−	−

Grade of changes: − none; ± minimum, + mild, ++ moderate, +++ remarked

#### Morphological features of alveolar macrophages by TEM (Fig. 5)

**Fig. 5:**
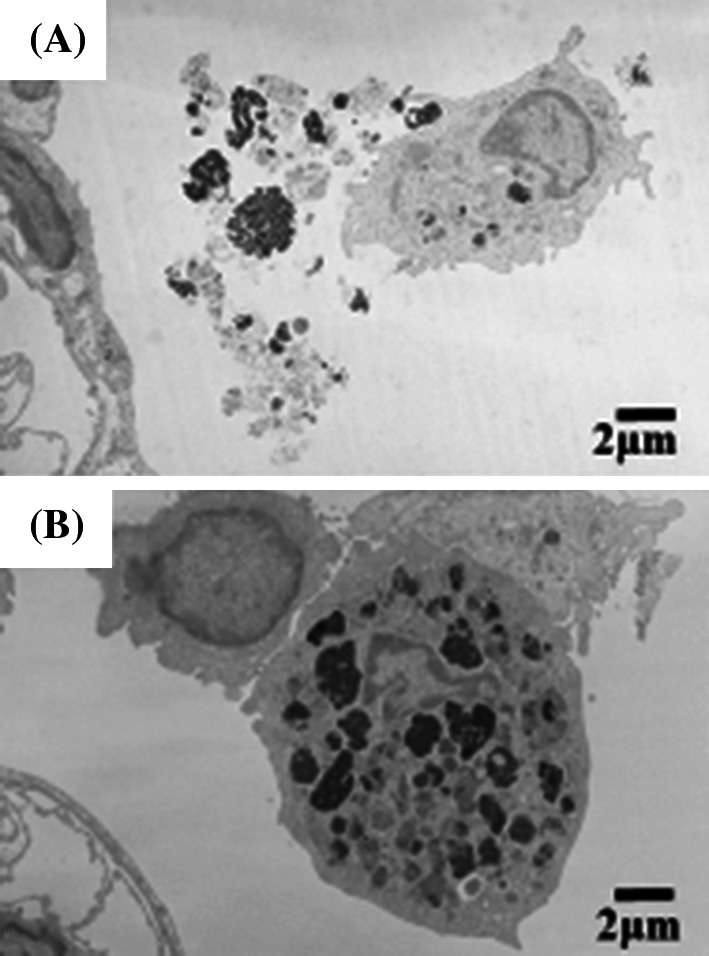
TEM images of lung tissue in the high-dose group at 3 days (**a**), and at 3 months (**b**) after CeO_2_ instillation, respectively. Agglomeration of CeO_2_ nanoparticles were observed in phagosomes of alveolar macrophages and some agglomeration of CeO_2_ nanoparticles were outside of cells. Agglomeration of CeO_2_ nanoparticles was also observed in the high-dose exposed group at 3 months following intratracheal instillation

Figure 5a shows TEM images of the alveolar space in the high-dose CeO_2_ instillation group lung tissue at 3 days after exposure. Black particle aggregates were observed with some cell organelles, so it seems that these were corpses of the alveolar macrophages that had taken up CeO_2_ nanoparticles. Macrophages that had taken up instilled CeO_2_ nanoparticles formed foam cells at 3 days after instillation. Figure 5b shows TEM images of alveolar macrophages in the high-dose CeO_2_ instillation group lung tissue at 3 months after exposure. Large amounts of black particles were observed in the cytoplasm. Some CeO_2_ still remained in the alveolar macrophages at 3 months after instillation.

### Inhalation study

#### Cell analysis in BALF

**Fig. 6:**
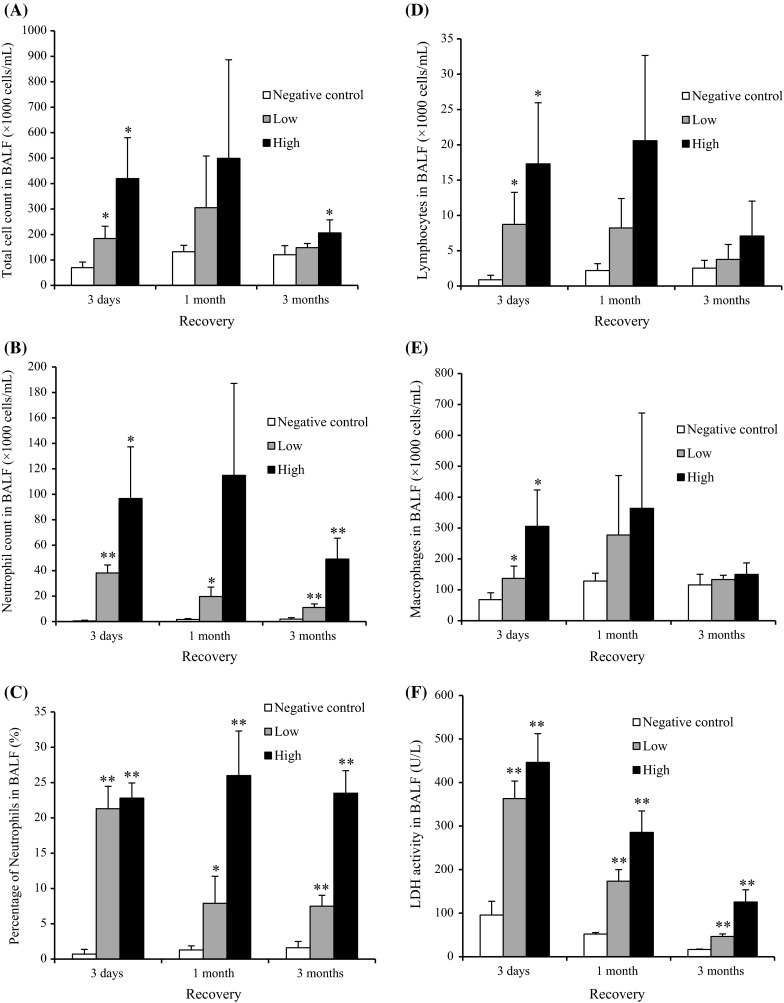

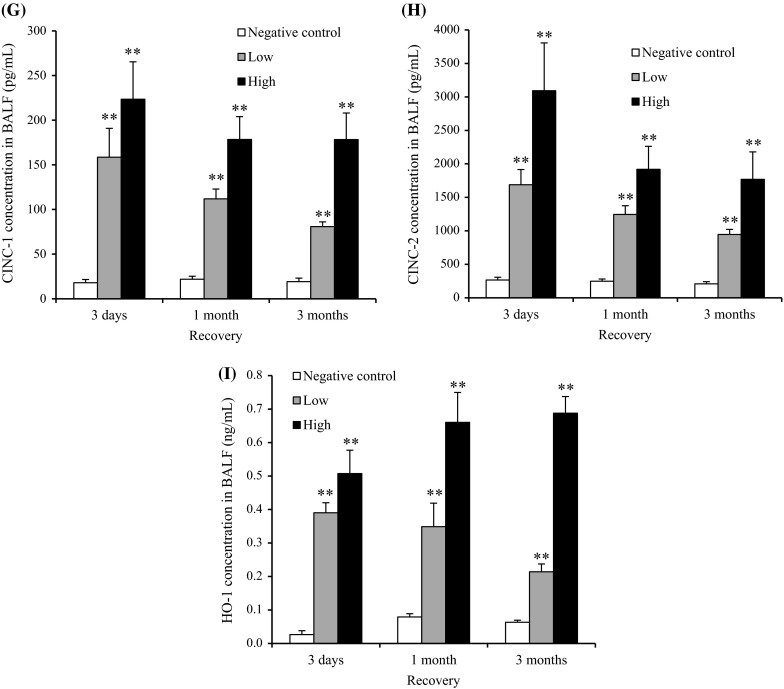
Cell analysis and cytokine concentration in BALF following inhalation of nanoparticles. **a** Total cell count in BALF. **b** Neutrophil count in BALF. **c** Percentage of neutrophils in BALF. **d** Lymphocyte count in BALF. **e** Alveolar macrophage count in BALF. **f** LDH activity in BALF. **g** Concentration of CINC-1 in BALF. **h** Concentration of CINC-2 in BALF. **i** Concentration of HO-1 in BALF. Inhaled CeO_2_ nanoparticles induced the influx of inflammatory cells such as neutrophils and expression of CINC-1, CINC-2, and HO-1 in BALF

Figure 6 shows the cellular analysis of the BALF following the inhalation of CeO_2_ nanoparticles. The total cell counts were dose-dependently high at the observation times, and the peak was at 1 month. The neutrophil counts and percentage in the BALF were persistently high in the low- and high-concentration groups during the observation times, except at 1 month in the high-concentration group. Although the macrophage and lymphocyte counts were elevated dose-dependently at 3 days postexposure, there were no significant differences between the unexposed and exposed groups thereafter. A persistent increase in the released LDH activity was observed in both concentration groups, and the peak of LDH level was at 3 days.

#### CINC concentration in BALF

Figure 6g–h shows the concentrations of CINC-1 and CINC-2 in the BALF following the inhalation of CeO_2_ nanoparticles. Both values in the low- and high-concentration groups were dose-dependently and significantly elevated during the observation time, although the values gradually decreased according to time.

#### HO-1 concentration in BALF

Figure 6i shows the concentration of HO-1 in the BALF following the inhalation of CeO_2_ nanoparticles. Compared with the unexposed group, the concentration of HO-1 in the low- and high-concentration groups was dose-dependently high during the observation time. The value in the low- and high-concentration groups decreased and increased according to time, respectively.

#### Histopathological changes in the lungs (Fig. 7, Table 3)

The distribution patterns of the inflammatory cells and macrophages were similar to those in the instillation groups. More macrophages were observed in the high-dose inhalation groups than in the low-dose inhalation groups. At 3 days after exposure, the enlarged macrophages contained a few brown particles, and the number of brown particles in the macrophages increased after 1 or 3 months of exposure.

**Fig. 7:**
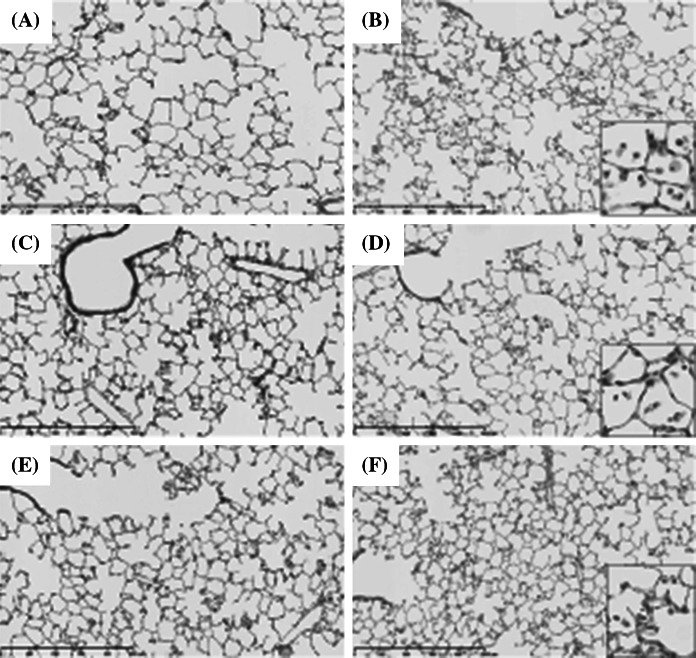
Hematoxylin and eosin staining of lung sections following inhalation of CeO_2_ nanoparticles. (×40, *inset* ×200). **a** Unexposed lung at 3 days, **b** CeO_2_-exposed lung (high concentration) at 3 days. **c** Unexposed lung at 1 month, **d** CeO_2_-exposed lung (high concentration) at 1 month. **e** Unexposed lung at 3 months, **f** CeO_2_-exposed lung (high concentration) at 3 months. Persistent inflammation, mainly neutrophils and alveolar macrophages, was observed after intratracheal instillation of CeO_2_ nanoparticles. Mild infiltration of neutrophils and alveolar macrophages in the lung was observed after 4 week’s inhalation of CeO_2_ nanoparticles

**Table 3:** Pathological features in the rat lung following inhalation of CeO_2_ nanoparticles

Pathological feature	3 days (*n* = 5)			1 month (*n* = 5)			3 months (*n* = 5)		
	Negative control	CeO_2_ low	CeO_2_ high	Negative control	CeO_2_ low	CeO_2_ high	Negative control	CeO_2_ low	CeO_2_ high
Macrophage infiltration in alveolar space	−	+	+	−	+	+	−	±	+
Neutrophil infiltration in alveolar space	−	−	−	−	−	−	−	−	±
Infiltration in interstitial area	−	−	−	−	−	+	−	−	−
Fibrosis	−	−	−	−	−	−	−	−	−
Tumor	−	−	−	−	−	−	−	−	−

Grade of changes: − none; ± minimum, + mild, ++ moderate; +++ remarked

Compared to the inhalation groups, the macrophages in the instillation groups were accompanied by more lymphocytic infiltrations. Changes in the number of intracytoplasmic particles were different between the instillation groups and the inhalation groups in a time course, with more particles being observed in the early periods in the instillation groups, and a higher number of particles in the late periods in the inhalation groups.

#### Morphological features of alveolar macrophages by TEM (Fig. 8)

**Fig. 8:**
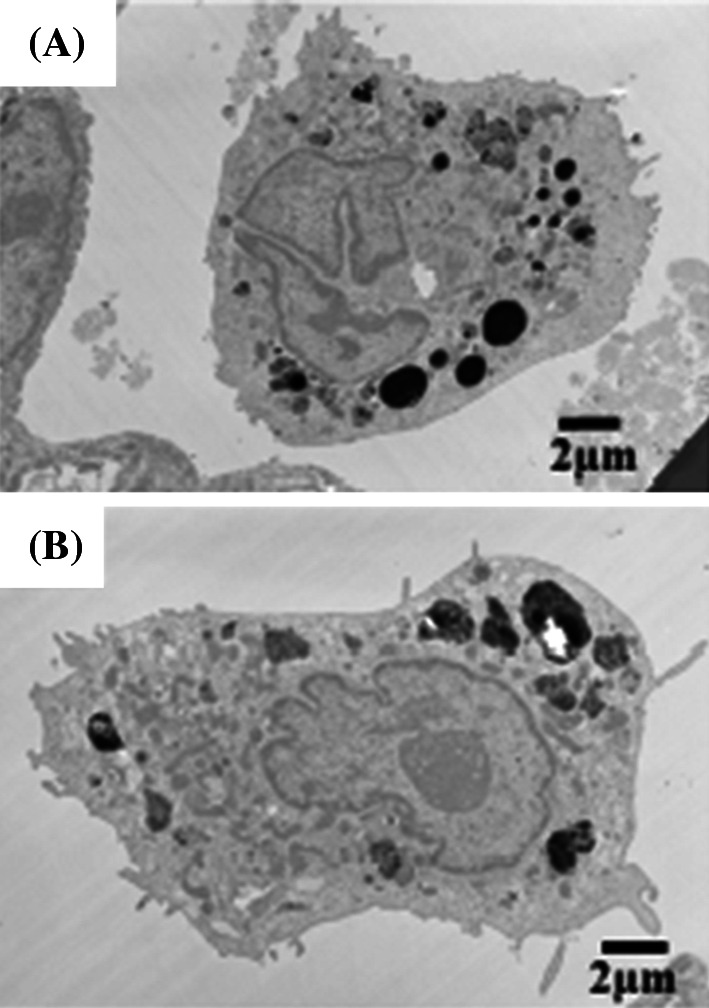
TEM images of lung tissue in the high-dose group at 3 days (**a**), and at 3 months (**b**) after CeO_2_ inhalation, respectively. Many phagosomes containing CeO_2_ nanoparticles were observed in alveolar macrophages. The amount of CeO_2_ nanoparticles in alveolar macrophages decreased according to time

A TEM image of alveolar macrophages in the high-dose CeO_2_ inhalation group lung tissue at 3 days after exposure is shown in Fig. 8a. Black contrasting objects of micron size were observed in the cytoplasm of alveolar macrophages. As large CeO_2_ aerosol of 1 μm was observed, as shown in Fig. 2b, these large black objects in the alveolar macrophages were thought to be CeO_2_ aerosol. Figure 8b shows TEM images of alveolar macrophages in the high-dose CeO_2_ inhalation group lung tissue at 3 months after exposure.

CeO_2_ aerosols were still observed in alveolar macrophages, and retention of CeO_2_ nanoparticles after 3 months was clarified.

## Discussion

In not only the inhalation but also in the intratracheal instillation studies, exposure to CeO_2_ induced persistent neutrophil inflammation in the rat lung, as revealed by cell analysis of BALF. If the initial lung burden of CeO_2_ following inhalation is calculated by the MPPD model (Kuempel et al. 2006), the initial lung burden in the low and high concentrations following 4 weeks of inhalation was 0.323 and 1.43 mg/rat (Data: Low concentration; CMD 0.087 μm (GSD 1.8) 2.09 mg/m^3^, High-concentration CMD 0.112 μm (GSD 1.83) 10.2 mg/m^3^), respectively. If the deposition rate (0.10035) of CeO_2_ in the lung is same between human and rat, the amounts of deposited CeO_2_ in rat lung at a concentration of 2 and 10 mg/m^3^ in the present experiment correspond to the amount that a worker is occupationally exposed to CeO_2_ at a concentration of 1 mg/m^3^ for 6 h/day, 5 days/week for 2.3, and 10.3 years, respectively (Human; lung weight 1000 g, tidal volume 625 ml, respiratory rate 12/min, Rat; lung weight 2 g). Considering the calculated values of the lung burden, the initial lung burdens in the low- and high-concentration groups in the inhalation study were similar to those in the low- and high-dose groups in the intratracheal instillation study, respectively. Our results showed that the neutrophil influx in the intratracheal instillation study was higher than that in the inhalation study. Some reports (Baisch et al. 2014; Silva et al. 2014; Morimoto et al. 2012) have shown the tendency that the infiltration of inflammatory cells following intratracheal instillation is superior to that following inhalation. According to Baisch et al. (2014), there were significantly more neutrophils in the BALF following the intratracheal instillation of TiO_2_ nanoparticles compared to inhalation, even though the initial lung burdens were the same. In our study (Morimoto et al. 2015), the pulmonary inflammation response following the intratracheal instillation of nickel oxide nanoparticles was equal to or higher than that following inhalation, in spite of the initial lung burden following the inhalation and intratracheal instillations being the same. These differences in pulmonary response between both studies may have been due to the bolus effect in the intratracheal instillation.

Previous reports (Aalapati et al. 2014; Ma et al. 2011; Gosens et al. 2014; Peng et al. 2014; Cho et al. 2010) have shown that exposure to CeO_2_ induces neutrophil inflammation in animal lung, although the observation periods in most of those studies were 1 month or less. Among them, some reports (Aalapati et al. 2014; Ma et al. 2011) showed that the inflammation level stayed the same, and some (Gosens et al. 2014; Peng et al. 2014; Cho et al. 2010) showed that the inflammation level decreased. Considering that persistent inflammation is an important endpoint which leads to irreversible lesion, such as lung tumor and fibrosis, it is thought that the inflammogenic effect of CeO_2_ on the lung is inconclusive. In observation period of 1 month, there are reports that even particles with low toxicity induced pulmonary inflammation following intratracheal instillation (Ogami et al. 2007; Bellmann et al. 2003).

We previously reported that exposure to fullerene and TiO_2_ nanoparticles with low toxicity induced significant neutrophil infiltration in rat lung until 3 or 6 months postexposure, although the level was mild (Morimoto et al. 2010; Oyabu et al. 2013). On the other hand, it has been reported that particles with high toxicity gradually induced pulmonary inflammation in a time-dependent fashion. Intratracheal exposure of nickel oxide nanoparticles induced pulmonary inflammation in rats, and the peak of inflammation was at 3 months postexposure. Sellamuthu et al. (2011) reported that when rats were exposed to inhalation of crystalline silica (15 mg/m^3^, 6 h/day, 5 days), pulmonary damage was determined after the latent periods (0–16 weeks). The number of neutrophils and the concentration of MCP-1 in the BALF were at the maximum after 16 weeks. Langley et al. (2004) conducted a 6-week inhalation of silica with 27 weeks of postexposure, and the number of neutrophils and lymphocytes in the BALF was high at 10 weeks postexposure, although not at 4 days, and the LDH and protein concentrations in the BALF were significantly high at 10 and 17 weeks, but not at 4 days.

In the present study, persistent inflammation in rat lung was observed in not only the acute phase but also in the chronic phase, such as 3 and/or 6 months postexposure, following both intratracheal instillation and inhalation, suggesting that CeO_2_ nanoparticles have inflammatory potential which may lead to irreversible lesion in the rat lung. Exposure to CeO_2_ induced the release of LDH at 3 days postexposure following not only inhalation but also intratracheal instillation. The level of LDH activity following the intratracheal instillation was higher than that following inhalation. This phenomenon, in which the stimulating effect of CeO_2_ following intratracheal instillation was more than that following inhalation, was also observed in the expression of HO-1, which is a representative oxidative stress marker. Therefore, it is possible that CeO_2_ induces lung injury through oxidative stress. Although it was not CeO_2_ nanoparticles, there was a report that ascorbic acid attenuated the acute pulmonary inflammation from exposure to metal oxide nanoparticles (Fukui et al. 2015).

We measured the concentrations of CINC-1 and 2, representative neutrophil chemotactic factors (Mitsuhashi et al. 1999; Hata et al. 2003), in the BALF and in the lung tissue in the present study. It is known that CINC-1 and 2 produced by macrophages have a function of activation and chemoattractant of neutrophils. Exposure to CeO_2_ persistently increased the concentration of CINC-1 and CINC-2 in the BALF following the intratracheal instillation and inhalation. We previously reported that particles with high inflammatory potentials induced a persistent gene expression of CINC, and that particles with low inflammatory potentials did not induce it or did so only transiently (Morimoto et al. 2010, 2015; Ogami et al. 2007). Intratracheally, nickel oxide nanoparticles also persistently increased the gene expression of CINC-1 and CINC-2 in lung tissue, accompanied by persistent neutrophil inflammation, regardless of the size of the agglomeration (Nishi et al. 2009). Titanium dioxide nanoparticles and fullerene caused a transient increase in the gene expression of CINC-1 and CINC-2 in lung tissue, as did CeO_2_, accompanied by transient neutrophil inflammation in intratracheal instillation studies, but did not cause gene expression of CINC-1 or CINC-2 or an influx of neutrophils in inhalation studies (Morimoto et al. 2015). As previous reports have shown a positive relationship between neutrophil influx in the lung and gene expression of CINC, it is suggested that the gene expression of CINC in the present study may be related to the neutrophil infiltration in the lung exposed to CeO_2_.

It has been reported that the activation of AP-1 and NF-kappaB as the transcription factor may induce the gene expression of CINC (Kaiboli et al. 2006; Eom and Choi 2009; Jia et al. 2007). Jia et al. reported that the activation of p38 induced the gene expression of CINC-1 through the activation of AP-1 and NF-kappaB in stress-induced gastric mucosal injury. Kaiboli et al. reported that HGF induced the gene expression of CINC-1 through activation of NF- kappaB in hepatocytes. On the other hand, Eom et al. reported that the oxidative stress induced by CeO_2_ nanoparticles induced signal pathway of ERK, P38, and JNK and the activation of NF- kappaB in human bronchial epithelial cells. From these reports, it is thought that the mechanism of lung inflammation by exposure to CeO_2_ is that the CeO_2_ nanoparticles may induce the gene expression of CINC through the activation of AP-1 and NF-kappaB.

In summary, we performed inhalation and intratracheal instillation of CeO_2_ nanoparticles in order to explore their toxicity. In the intratracheal instillation study, F344 rats were exposed to 0.2 or 1 mg of CeO_2_ nanoparticles. In the inhalation study, rats were exposed to the maximum concentration of inhaled CeO_2_ nanoparticles for 4 weeks. The intratracheal instillation of CeO_2_ nanoparticles caused a persistent increase in neutrophil influx in the lung, and high concentrations of CINC-1, CINC-2, and HO-1. The inhalation of CeO_2_ nanoparticles also induced a persistent influx of neutrophils and expression of CINC-1, CINC-2, and HO-1 in BALF. Taken together, CeO_2_ nanoparticles may have a pulmonary toxicity which can lead to irreversible lesion.
